# Supplementary material for: MSX1—A Potential Marker for Uterus-Preserving Therapy of Endometrial Carcinomas
Source: Int J Mol Sci. 2020 Jun 25;21(12):4529. doi: 10.3390/ijms21124529 (PMC7350265; doi:10.3390/ijms21124529)

Fig. 1 Kaplan Meier overall survival curve in terms of IRS (p=0.41)

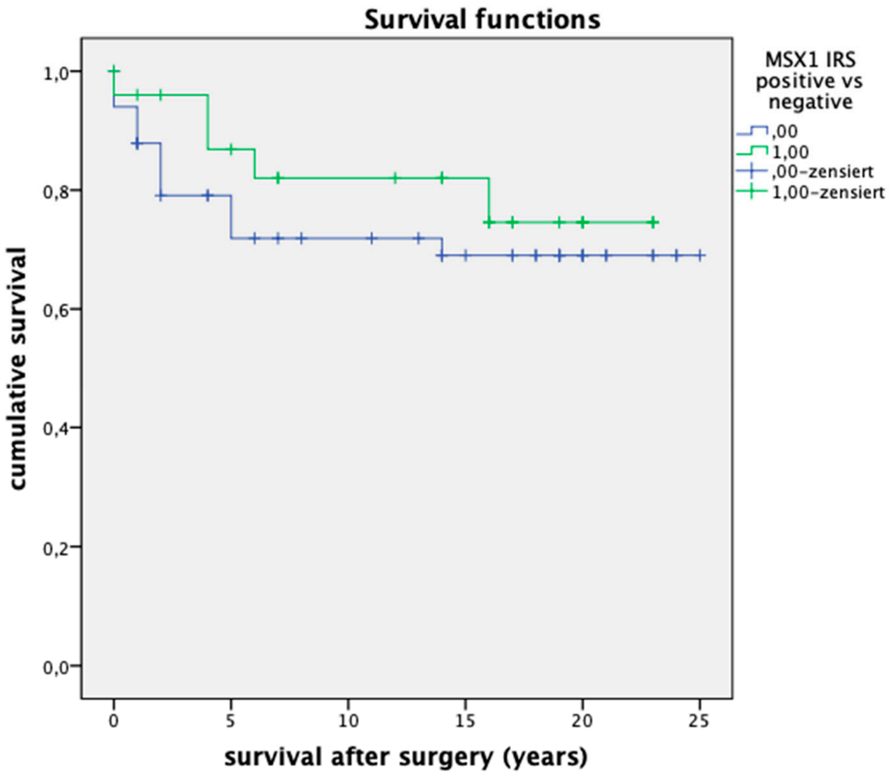

Fig. 2 Kaplan Meier overall survival curve in terms of IRS, Grading 1 (p=0.862)

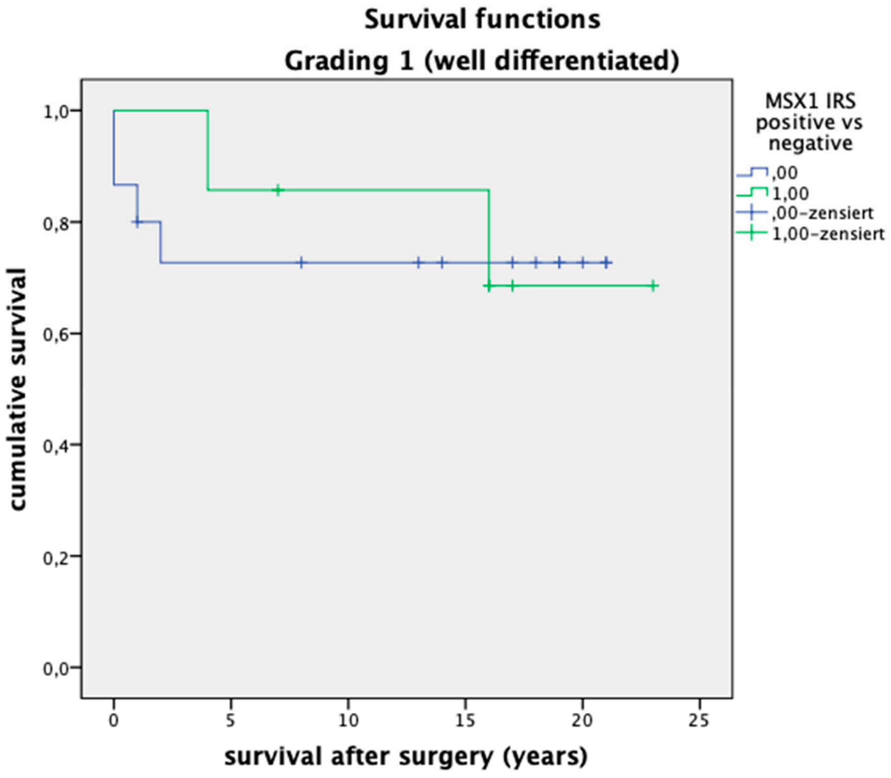

Fig. 3 Kaplan Meier overall survival curve in terms of IRS, Grading 2 (p=0.546)

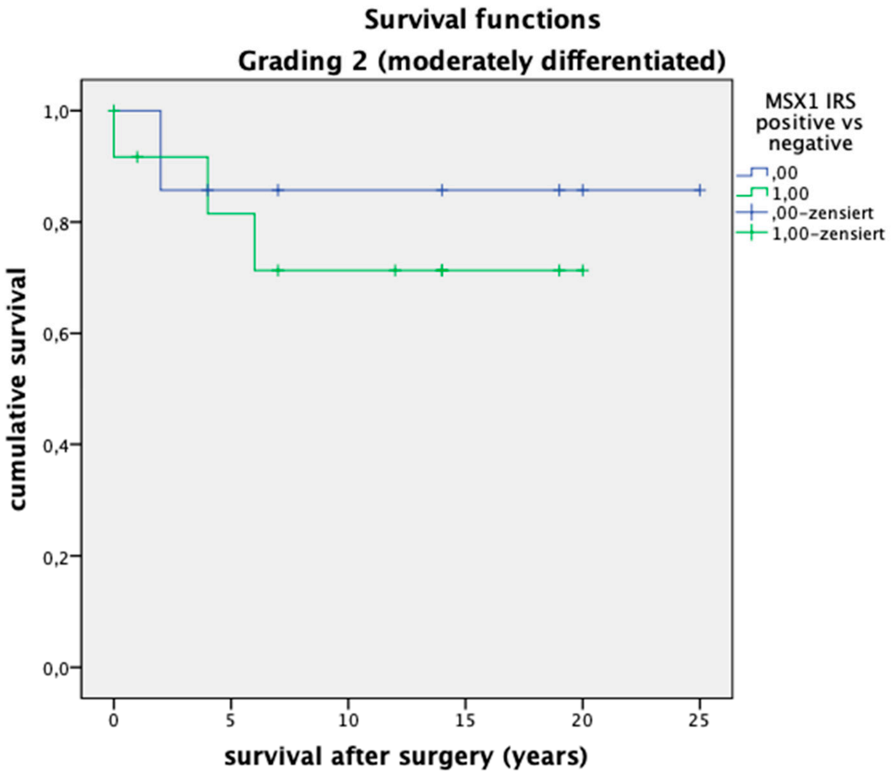

Fig. 4 Kaplan Meier overall survival curve in terms of IRS, Grading 3 (p=0.116)

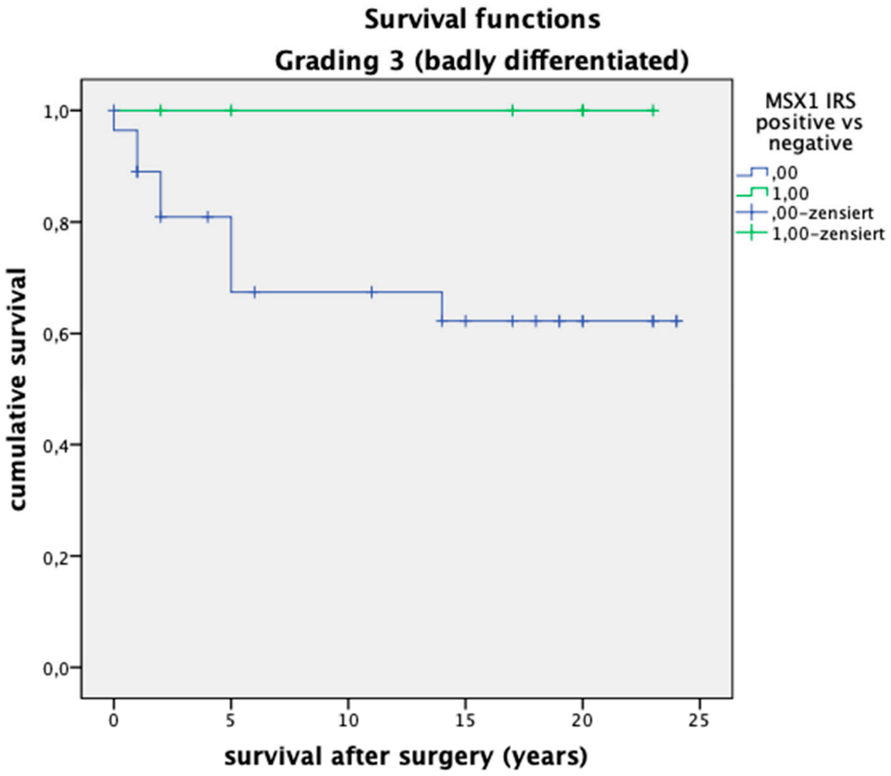

Kaplan Meier overall survival curves for endometrial and ovarian carcinomas.

Fig. 5 Endometrial carcinomas (p=0.116)

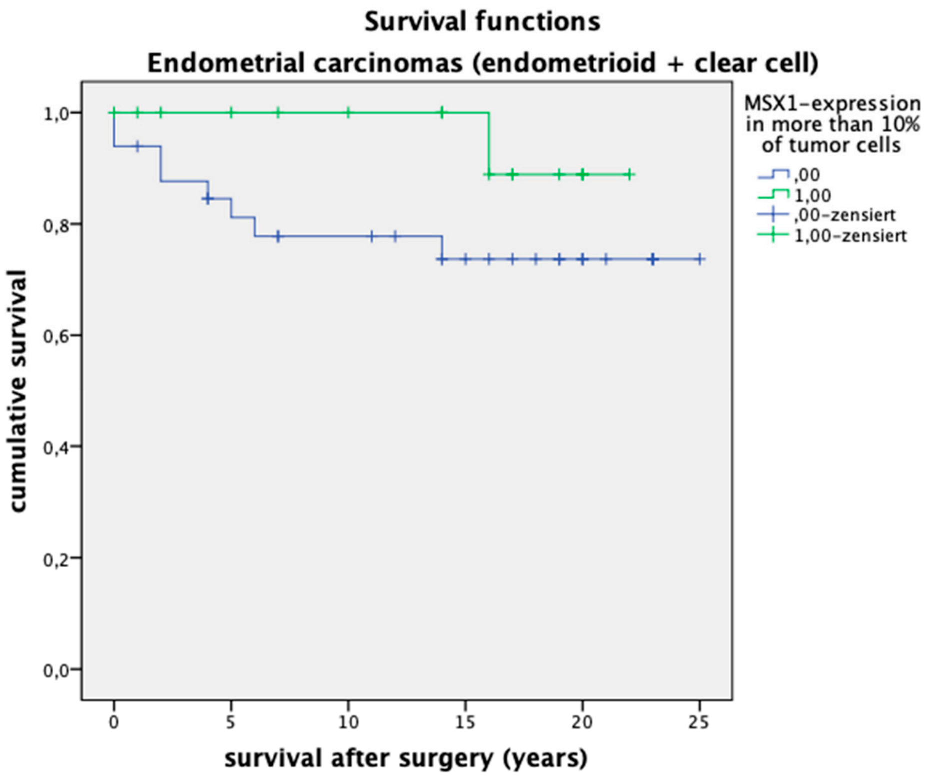

Fig. 6 Ovarian carcinomas (p=0.414)

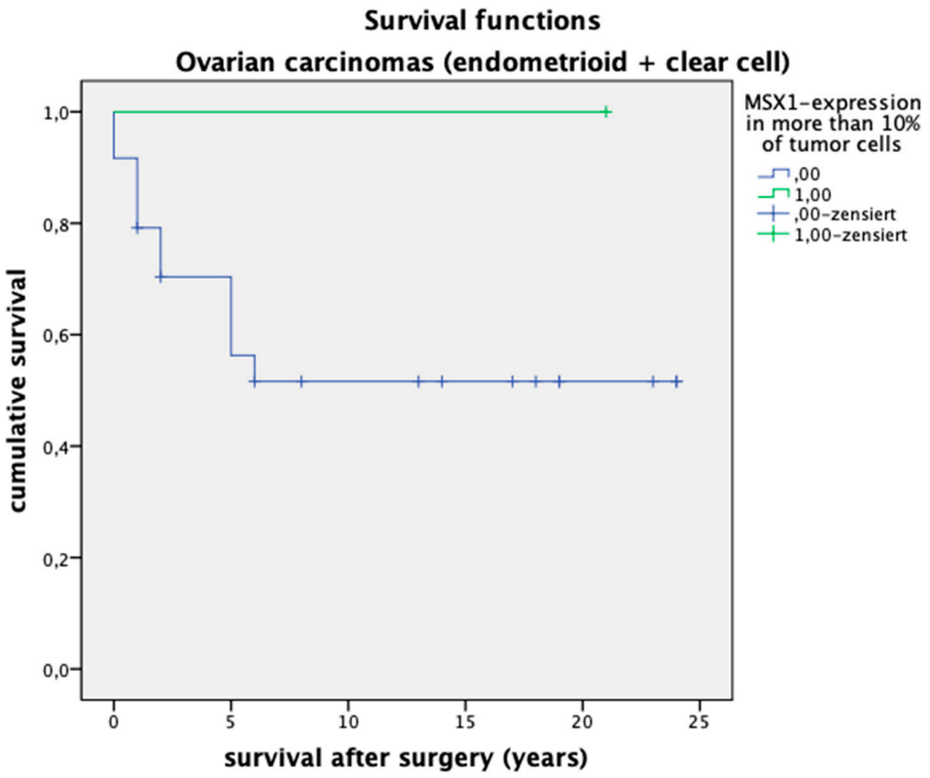

Kaplan Meier overall survival curves for grading.

Fig. 7 Graded 1 tumors (well differentiated) (p=0.404)

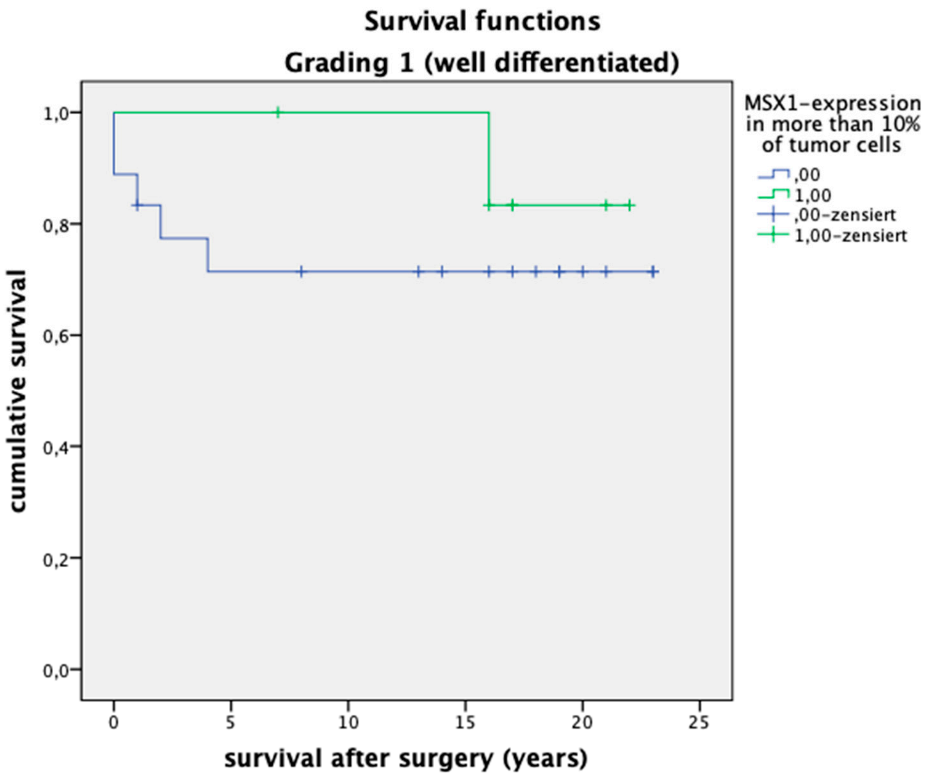

Fig. 8 Graded 2 tumors (moderately differentiated) (p=0.135)

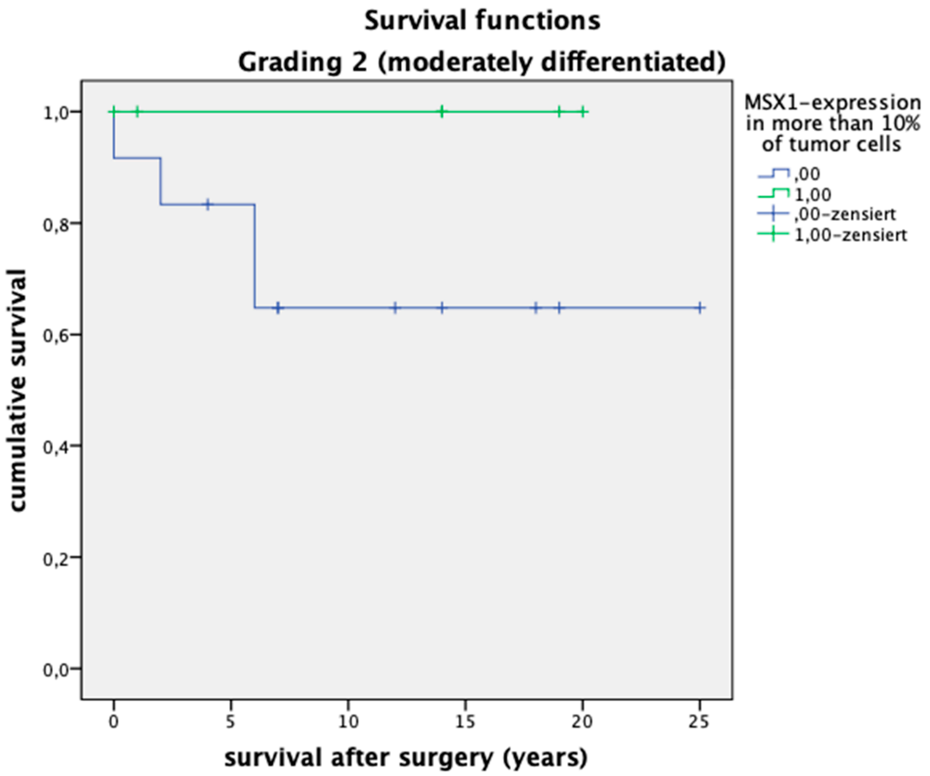

Fig. 9 Graded 3 tumors (badly differentiated) (p=0.142)

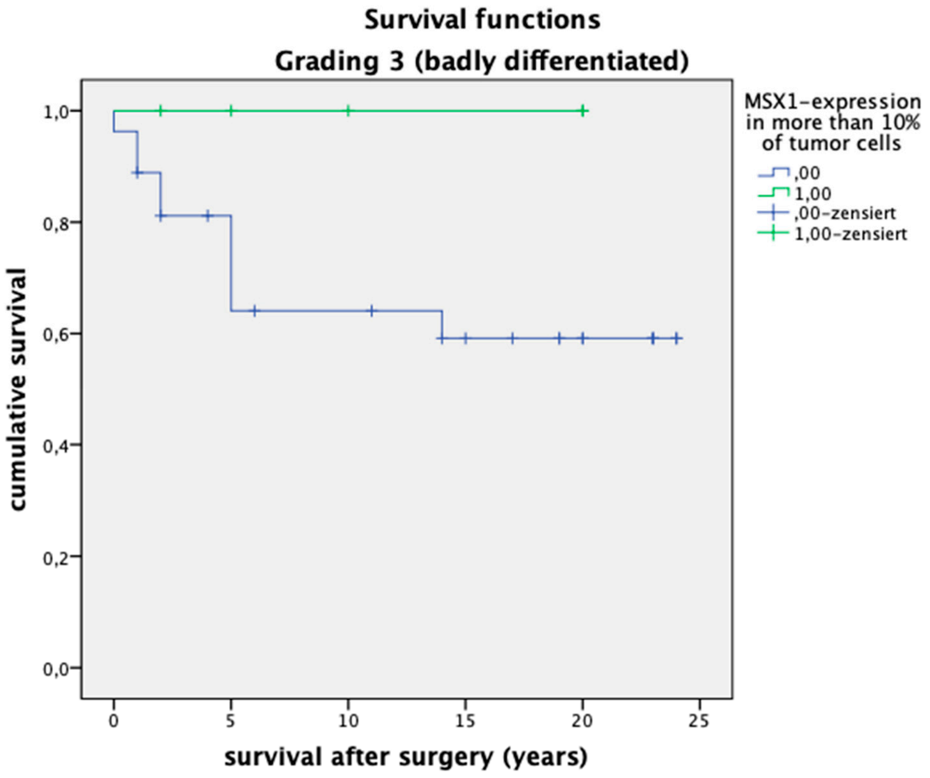

Fig. 10 ROC curve (IRS, p=0.619)

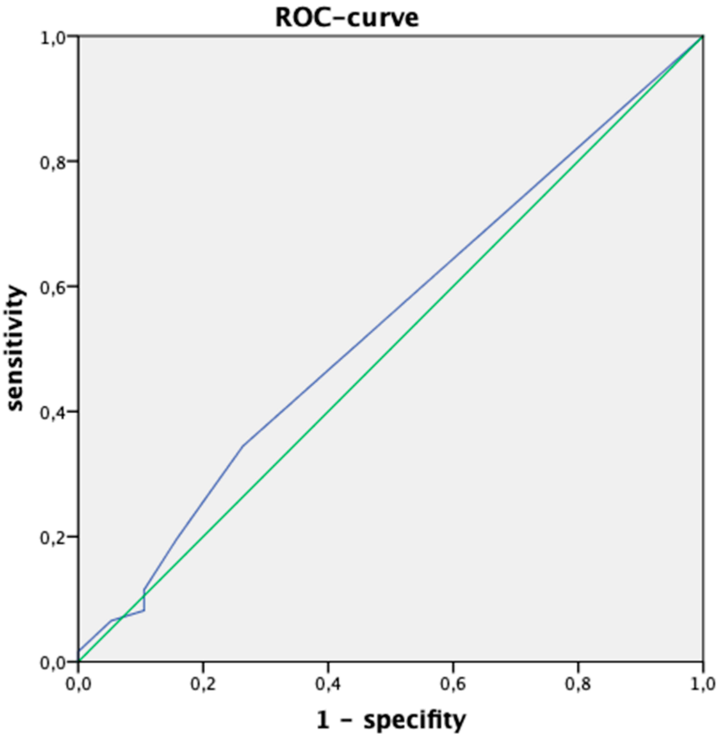

Fig. 11 ROC curve (percentage of MSX1 expression in tumor cells, p=0.591)

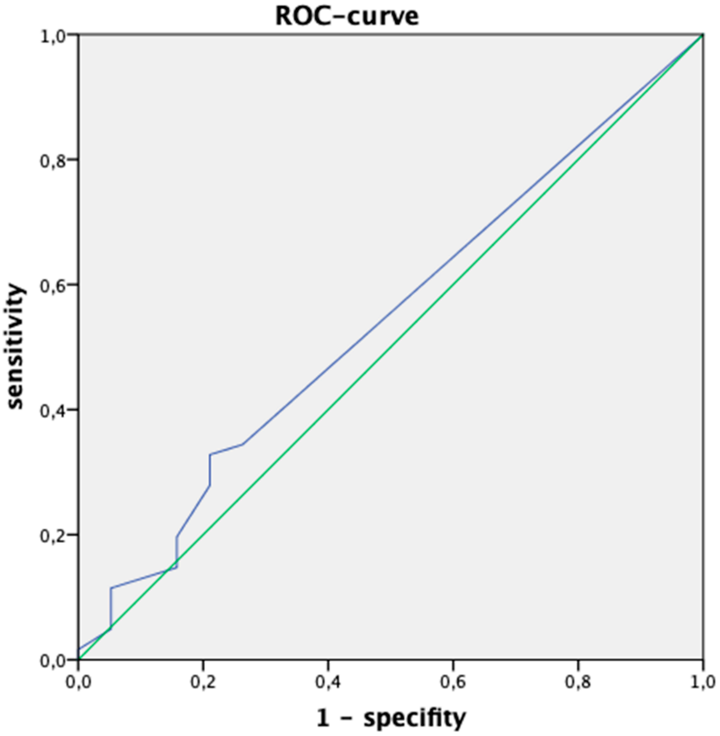

Supplement: Supplementary file 1 [file ijms-21-04529-s001.pdf]
